# (Dis-)Empowering Motivational Climate and Social Physique Anxiety in Physical Education: Is There a Gender Difference?

**DOI:** 10.3390/bs16091686

**Published:** 2026-09-19

**Authors:** Antonio Granero-Gallegos, Luis M. Salmerón-Hinojosa, María Carrasco-Poyatos, Ginés D. López-García

**Affiliations:** 1 Faculty of Education and Health Research Center, University of Almeria, 04120 Almeria, Spain; agranero@ual.es (A.G.-G.); lsh076@inlumine.ual.es (L.M.S.-H.); carrasco@ual.es (M.C.-P.); 2 Higher Institute of Teaching University Centre, University of Murcia, 30003 Murcia, Spain

**Keywords:** body image, dark side, negative self-esteem, gender, SPA

## Abstract

Background: Building on Duda’s hierarchical approach, this study examined gender differences in students’ perceptions of (dis-)empowering motivational climates, self-esteem and social physique anxiety in the physical education context. Furthermore, it analysed the associations of (dis-)empowering climates with these self-presentation-related outcomes among boys and girls. Methods: A total of 2438 Spanish Physical Education students (Mage = 16.15 ± 2.01; 50.74% girls) participated in this cross-sectional research. A multi-group structural equation modeling approach was used to answer the research objectives. Results: Girls scored significantly higher than boys in negative self-esteem and social physique anxiety. The multi-group structural equation modeling showed slightly higher coefficients among boys for the associations between empowering climate and positive self-esteem and between disempowering climate and negative self-esteem. The negative self-esteem–SPA coefficient was higher among boys, whereas the inverse positive self-esteem–SPA coefficient was higher among girls. Conclusions: The main findings underscore the relevance of PE teachers creating empowering climates and avoiding disempowering climates, given their associations with positive self-esteem and social physique anxiety among boys and girls.

## 1. Introduction

During adolescence, individuals may develop heightened concerns regarding how others evaluate their physical appearance (Hausenblas et al., 2004). This preoccupation is often influenced by bodily changes associated with growth and maturation, such as height, weight, or body mass index (BMI). Specifically, BMI reflects fluctuations in body composition throughout the developmental process, which may lead adolescents to become excessively concerned about external judgments of their physique, whether from peers, family members, or others, potentially resulting in social physique anxiety (SPA; Hart et al., 1989). A substantial body of research highlights the key role of the teacher in fostering a healthy self-presentation among students. Teachers influence students not only through their verbal and non-verbal behaviors, but also by how they structure and manage the physical education (PE) classroom environment, shaping what is commonly referred to as the PE motivational climate (Appleton & Duda, 2016). Nevertheless, although emerging evidence underscores the relevance of (dis-)empowering climates in shaping students’ self-regulation and behavioral outcomes in PE, limited attention has been given to how students’ perceptions of teacher-created empowering or disempowering climates may contribute to the development of SPA during PE classes. Furthermore, the mediating role of self-esteem in this relationship remains underexplored.

On the other hand, gender differences have been identified in self-presentation variables within the context of physical education (Hagger & Stevenson, 2010). Given that girls tend to report higher levels of social physique anxiety (SPA) than boys in physical activity settings (Hagger & Stevenson, 2010; Huang et al., 2024; Rojo-Ramos et al., 2024b), future research should adopt a gender-sensitive approach when examining the relationship between the motivational climate and students’ self-presentation outcomes, such as SPA and self-esteem, in PE contexts. Due to the lack of previous research, it is essential to consider gender when analyzing the associations between teacher-created empowering and disempowering climates and students’ self-presentation during PE lessons. This may help identify how (dis-)empowering climates are associated with self-presentation outcomes in boys and girls and inform efforts to discourage disempowering and promote empowering climates in PE. Grounded in Duda’s (2013) theoretical framework, the present study aims to extend previous evidence by examining gender differences in the relationships between teacher-created (dis-)empowering climates and students’ self-presentation outcomes in physical education.

### 1.1. Social Physique Anxiety in Physical Education

In the field of physical activity, self-presentation has primarily been studied in terms of SPA (Hausenblas et al., 2004). SPA has been conceptualized as an affective response reflecting individuals’ concerns about how their bodies may be judged by others (e.g., height, weight, or muscular tone) (Hausenblas et al., 2004). In this regard, students with higher levels of SPA are more likely to worry that their physical appearance will be negatively evaluated in such contexts. Consequently, they tend to avoid situations that may lead to bodily exposure, such as PE lessons, and may adopt strategies such as wearing loose-fitting clothes or choosing positions in the classroom where they feel less observed (Sabiston et al., 2021; Liardi et al., 2023). Furthermore, there is a substantial body of research that has related SPA to salient psychological and behavioural factors in the PE context, such as self-esteem (Hagger & Stevenson, 2010), motivation (Sicilia et al., 2014), and intention to be physically active (Sicilia et al., 2016), and contextual factors, such as motivational climate (Miller & Fry, 2018).

### 1.2. Self-Esteem

SPA is closely allied with another self-related construct, self-esteem (Moradi et al., 2021). Self-esteem represents an individual’s perception of the self in physical contexts and has descriptive and evaluative content. In this regard, students focused on self-presentation tend to manage their impressions in contexts where they anticipate being evaluated by others (Leary & Kowalski, 1990). Those with high self-esteem will feel secure about their appearance in PE lessons, are not likely to be concerned about how their physique is perceived and, as a result, will show lower levels of SPA (Hagger & Stevenson, 2010). Conversely, students with lower levels of self-esteem are likely motivated to avoid displaying their physique in a way they believe others will perceive negatively, which may be associated with higher levels of SPA (Hagger & Stevenson, 2010). From this perspective, Rosenberg (1965) proposed that self-esteem arises as a consequence of social comparison processes, whereby individuals assess themselves either positively (i.e., positive self-esteem) or negatively (i.e., negative self-esteem). Previous literature has demonstrated that self-esteem is inherently influenced by the perceived evaluation of one’s social group, highlighting the central role of the teacher in PE settings (Granero-Gallegos et al., 2023a; Valero-Valenzuela et al., 2019). Indeed, recent studies have examined how teaching styles adopted by PE teachers are associated with positive and negative self-esteem among students. However, these studies, despite being based on Self-Determination Theory (SDT; Ryan & Deci, 2017), differ from the multidimensional conceptualisation proposed in Duda’s theoretical framework. Therefore, adding evidence of how (dis-)empowering climates are associated with students’ self-presentation in PE classrooms represents an important contribution.

### 1.3. Motivational Climates in Physical Education

Recent work has highlighted classroom climate as a central antecedent of students’ learning-related behaviors, motivation, and active engagement during PE lessons (White et al., 2021). Building on this idea, Appleton and Duda (2016) define motivational climate through a hierarchical, multidimensional lens that brings together core features of the social environment described in Self-Determination Theory (Ryan & Deci, 2017) (autonomy support, control, and social support) and in Achievement Goal Theory (Ames, 1992) (task-involving and ego-involving climates). From this integrated perspective, PE teachers can cultivate climates that are relatively more empowering or more disempowering, thereby strengthening or undermining students’ experiences and outcomes in class (Appleton & Duda, 2016; Granero-Gallegos et al., 2023b; Espinoza-Gutiérrez et al., 2024). An empowering climate is characterised by teaching practices that acknowledge students’ views and preferences (autonomy support), emphasise improvement and effort as standards for success (task-involving), and communicate care and respect for students as individuals (social support). In contrast, a disempowering climate is reflected in coercive or pressuring strategies aimed at shaping how students think, feel, or behave (control), alongside success criteria grounded in social comparison and demonstrations of superiority (ego-involving cues).

Appleton and Duda (2016) further argue that empowering climates may protect students from maladaptive experiences, whereas disempowering climates may increase the likelihood of negative outcomes during PE. Consistent with these propositions, studies applying this framework have linked empowering climates with higher enjoyment and concentration, while disempowering climates have been associated with greater state and trait anxiety in PE settings (Becerra-Fernández et al., 2025; Milton et al., 2025; Simon et al., 2025). Despite these advances, research has not yet directly tested how empowering and disempowering climates relate to self-presentation outcomes in PE. The only study identified (Miller & Fry, 2018) did not adopt the (dis-)empowering climate conceptualisation and did not examine body esteem and social physique anxiety (SPA) as outcomes of classroom climate, which prevents conclusions about the association of motivational climate with self-presentation processes in PE. Accordingly, there remains a clear need for research that, grounded in the empowering versus disempowering distinction and informed by a gender perspective, examines how the motivational climate created by PE teachers is associated with SPA among PE students.

### 1.4. Gender Differences

In the PE context, previous research has reported mixed findings regarding gender differences in students’ perceptions of teacher-created motivational climate and self-presentation outcomes. For example, some studies have reported no significant gender differences in students’ perceptions of motivational climate (Burgueño et al., 2024), self-esteem (Granero-Gallegos et al., 2023a), or SPA (Sáenz-Alvarez et al., 2013). In contrast, other studies have found that girls tend to report higher levels of SPA (Hagger & Stevenson, 2010) and lower levels of self-esteem (Krutsevych & Marchenko, 2017). However, to date, no study has examined gender differences in the perception of (dis-)empowering climates in PE settings. From a theoretical perspective, examining gender differences in relation to motivational climates within the theoretical framework proposed by Duda (2013) can enhance our understanding of these disparities between girls and boys in PE contexts. To obtain better insight into the associations of (dis-)empowering climates with boys’ and girls’ self-presentation experiences in PE, additional research is required.

### 1.5. The Present Study

To fill the aforementioned gaps in the literature, the objective of this research was to examine possible gender differences in the associations among teacher-created empowering and disempowering climates, self-esteem, and SPA. To this end, we first examined the possible gender differences in terms of teacher-created (dis-)empowering climates, self-esteem and SPA. Second, a multi-group structural equation modeling analysis was conducted to explore the structural associations among motivational climates, self-esteem, and SPA across boys and girls. Given the nascent state of research in this specific area, this study was conducted without the formulation of prior hypotheses. Reporting followed the Strengthening of the Reporting of Observational Studies in Epidemiology (STROBE) Initiative (Sánchez-Martín et al., 2024; Von Elm et al., 2007).

## 2. Materials and Methods

### 2.1. Study Design

The present study employed an observational, cross-sectional design, involving secondary school students from various educational institutions (high schools) in Spain. Inclusion criteria were (i) active enrollment in a Spanish secondary education center within the subject of PE; (ii) participation in PE courses corresponding to the 3rd or 4th year of compulsory secondary education or the baccalaureate, or enrollment in a medium- or higher-level training program specializing in PE and Sports; and (iii) regular in-person class attendance. The exclusion criteria included (i) lack of informed consent for the use of study data and (ii) incomplete or incorrectly filled data collection forms.

### 2.2. Sample Size

The Free Statistics Calculator v.4.0 software (Soper, 2025) was used to perform an a priori statistical power analysis for the appropriate sample size. For a structural equation model (SEM) with five latent variables and 22 observable variables, it was estimated that a minimum sample of 1171 participants would be required to detect an effect size of f^2^ = 0.13 with a 90% statistical power level and a significance level of α = 0.05. Furthermore, 2342 participants would be required to conduct a multigroup SEM analysis by gender (boys, girls). A total of 2438 students participated in the study.

### 2.3. Instruments and Measures

#### 2.3.1. Motivational Climate

The Spanish version of the Empowering and Disempowering Motivational Climate Questionnaire in Physical Education (EDMCQ-PE; Milton et al., 2018), adapted to the Spanish context by Granero-Gallegos et al. (2026), was employed in this study. The EDMCQ-PE is comparable to other instruments assessing perceived autonomy support and controlling styles. However, while it has been validated as a two-dimensional measure (empowering/disempowering), its items cover a broader range, including autonomy support, task-involving, and social support (i.e., empowering), as well as control and ego-involving (disempowering). Furthermore, previous research has established its validity and reliability as a higher-order model (Granero-Gallegos et al., 2026). This instrument consists of 34 items, with 17 assessing an empowering motivational climate (e.g., “My teacher encouraged pupils to try new skills”) and 17 evaluating a disempowering motivational climate (e.g., “My teacher was less friendly with pupils if they didn’t make the effort to see things his or her way”). Based on the psychometric analysis conducted in this same sample (Granero-Gallegos et al., 2026), items 5, 15, 20, and 29 of the disempowering dimension were removed because they did not meet the predefined ESEM loading criteria. The resulting 30-item solution showed satisfactory factorial fit (CFI = 0.938; TLI = 0.927; RMSEA = 0.047; SRMR = 0.029). Consequently, the final version comprised 30 items, with 17 measuring empowering and 13 assessing disempowering motivational climates. In the present study, the internal consistency reliability was satisfactory: empowering, ω = 0.92; disempowering, ω = 0.83.

#### 2.3.2. Self-Esteem Scale

The Spanish adaptation of the Rosenberg Self-Esteem Scale (RSES), developed by Atienza et al. (2000) based on the original instrument by Rosenberg (1965), was utilized in this study. The RSES comprises 10 items designed to evaluate global self-esteem, with five items measuring positive self-esteem (e.g., “On the whole, I am satisfied with myself”) and five assessing negative self-esteem (e.g., “At times I think I am not good at all”). Responses were recorded using a Likert-type scale ranging from 1 (strongly disagree) to 4 (strongly agree). In the present study, the internal consistency obtained was satisfactory: positive self-esteem, ω = 0.76; negative self-esteem, ω = 0.84.

#### 2.3.3. Social Physique Anxiety

The Spanish version of the Social Physique Anxiety Scale (SPAS-7; Sáenz-Alvarez et al., 2013), originally developed by Motl and Conroy (2000, 2001), was used. This instrument consists of seven items that are grouped into a single factor and assess social anxiety related to physical appearance (e.g., “In the presence of others‚ I feel apprehensive about my physique or figure”). Responses were recorded using a Likert-type scale ranging from 1 (never) to 5 (always). In the present study, the internal consistency obtained was satisfactory, ω = 0.90.

#### 2.3.4. Demographics

Age, gender, height, and weight were self-reported by the participants. BMI was calculated as weight in kilograms divided by height in metres squared (kg/m^2^).

### 2.4. Procedure

After obtaining authorisation from the school’s management to carry out the data collection, the students were informed of the research’s purpose and importance, their rights as participants, the way to complete each scale, that the answers were anonymous, that they would not affect any grade in any way, and that they could stop participating at any time. The data collection was performed by a researcher during the PE lesson period. All individuals involved were clearly informed about the purpose of the study, the voluntary nature of their involvement, the confidentiality of their responses, and how the collected data would be handled. Participation was voluntary. Parent or legal-guardian informed consent and student assent were required before participation. Furthermore, according to Article 9 of Regulation (EU) 2016/679, better known as the General Data Protection Regulation (GDPR), ethical approval is not necessary because no sensitive data were collected during this study. Sensitive data in this context refers to information revealing racial or ethnic origin, political opinions, religious or philosophical beliefs, union membership, genetic data, biometric identifiers, or data concerning health, sexual life, or sexual orientation.

### 2.5. Risk of Bias

It is important to note that the sampling method employed was convenience sampling, with no randomization of participants. Blinding was maintained between the participants and the researchers responsible for data processing and analysis. In terms of selection bias, participation in the study was entirely voluntary, and all communication with students was conducted face-to-face.

### 2.6. Data Analysis

#### 2.6.1. Preliminary Data Analysis

Descriptive statistics were calculated for each study variable. In addition, latent correlations between variables were estimated, considering that values up to 0.85 indicate the absence of multicollinearity between the variables analyzed (Kline, 2023). The reliability of the scales was assessed using McDonald’s omega coefficient (ω), with values >0.70 considered indicative of adequate internal consistency (Hair et al., 2018). To verify univariate normality, standardized skewness and kurtosis values were analyzed, ranging from −0.65 to 0.60 for skewness and from −0.84 to 0.27 for kurtosis, thus supporting the assumption of normality (Kline, 2023). Subsequently, independent samples *t*-tests were conducted to examine potential gender-based differences (i.e., boys and girls) in (dis-)empowering motivational climate, self-esteem, and SPA. Levene’s test was used to test for homogeneity of variance. Effect sizes (ESs) were interpreted according to Cohen (1988): d-values of 0.2, 0.5, and 0.8 indicated small, medium, and large effects, respectively (Rodríguez-Sabiote et al., 2025). All statistical analyses were performed with JASP v.0.19.1.0, Department of Psychological Methods, University of Amsterdam (Amsterdam, Netherlands).

#### 2.6.2. Main Data Analysis

Associations among (dis-)empowering climates, positive and negative self-esteem, and SPA were examined using multigroup structural equation modelling following a two-step approach (Kline, 2023). The analysis was conducted in IBM SPSS Amos v.30, IBM Corporation (Armonk, NY, USA), using maximum likelihood (ML) estimation. Although the questionnaire responses were collected using Likert-type scales, they were treated as continuous variables in the SEM. The model comprised five focal latent constructs represented by 22 indicators: empowering climate was represented by autonomy support, task-involving climate, and social support; disempowering climate by control and ego-involving climate; positive and negative self-esteem were each represented by their five respective items; and SPA by its seven items. No item parceling was applied. First, the measurement model was evaluated. Measurement invariance across gender was subsequently assessed following the sequential procedure proposed by Milfont and Fischer (2010), involving four increasingly constrained models: configural invariance (unconstrained model), metric invariance (equal factor loadings), scalar or strong invariance (equal factor loadings and intercepts), and strict invariance (equal factor loadings, intercepts, and residual variances). Nested models were compared using changes in model-fit indices, with an increase in RMSEA of ≥0.015 or a decrease in CFI or TLI of ≥0.010 considered indicative of a lack of invariance (Chen, 2007). Second, the structural model estimated direct and cross-sectional indirect associations from motivational climates to SPA through self-esteem. Bootstrap procedures with 5000 resamples were used to estimate the confidence intervals of the indirect associations. Age, BMI, and educational institution were included as covariates. To control potential confounding effects arising from the nested structure of the data (students clustered within educational centers), a preliminary analysis of the Intraclass Correlation Coefficient (ICC) was conducted using linear mixed models in SPSS for all endogenous variables. The analysis yielded marginal values for each construct: Positive Self-Esteem (ICC = 0.042), Negative Self-Esteem (ICC = 0.033), and SPAS (ICC = 0.047). Given that all coefficients fall below the critical threshold of 0.05 recommended by Hox (2010), the residual variance due to clustering is statistically negligible. Consequently, incorporating the school variable as a control covariate in the AMOS SEM framework provides a robust and sufficient approach to ensure the validity of the structural estimates. The SEM was evaluated, taking into account the following goodness-of-fit: the chi-square ratio/degrees of freedom (χ^2^/df), CFI (Comparative Fit Index), TLI (Tucker-Lewis Index), RMSEA (Root Mean Square Error of Approximation) with its 90% confidence interval (CI), and SRMR (Standardized Root Mean Square Residual). For the χ^2^/df ratio, values < 5.0 are considered acceptable (Hu & Bentler, 1999), while CFI and TLI values > 0.95, RMSEA < 0.06 and SRMR < 0.05 are considered excellent goodness-of-fit indices for the model (Hooper et al., 2008; Hu & Bentler, 1998). CFI and TLI values between 0.90 and 0.95, and RMSEA and SRMR < 0.08, are considered acceptable goodness-of-fit indices for the model (Hooper et al., 2008; Marsh et al., 2004). According to Hayes (2017), a cross-sectional indirect association was considered statistically significant (*p* < 0.05) when its 95% confidence interval (CI95%) does not include zero. Finally, following Dominguez-Lara (2017), the explained variance (R^2^) was considered as ES, considering values <0.02 small, close to 0.13 medium, and >0.26 large (Cohen, 1988). The CI(95%) of R^2^ was also used to ensure that no value is less than the minimum required for interpretation (0.02).

## 3. Results

### 3.1. Preliminary Results

#### 3.1.1. Participants

A total of 2438 secondary education students participated in the study (48.32% boys, 50.74% girls, and 0.94% who did not specify their gender), with ages ranging from 14 to 21 years (*M* = 16.15; *SD* = 2.01). A total of 27 individuals did not provide consent for data collection and were therefore excluded from the study. Additionally, 128 questionnaires were discarded due to non-compliance with inclusion criteria or incomplete or incorrectly filled responses. Data collection was conducted between April and May 2025. No missing values were recorded in the final analyzed sample.

#### 3.1.2. Descriptive Statistics, Differences by Gender, and Latent Correlations

Table 1 presents the descriptive statistics of the different variables, considering the gender of the students, together with the results of the Student’s *t*-test for independent samples. For this analysis, the 23 participants who did not report their gender were excluded. Statistically significant differences were observed in all the variables studied. Boys reported significantly higher mean scores than girls in empowering, disempowering, and positive self-esteem. On the other hand, girls reported significantly higher mean scores in negative self-esteem and SPA, with a moderate effect size for SPA. In addition, positive correlations between negative self-esteem and SPA were observed, both in boys and girls. Likewise, negative correlations between empowering and disempowering, as well as between positive self-esteem and negative self-esteem and SPA, were also observed.

### 3.2. Main Results

#### Paths from (Dis-)Empowering Climates to Social Physique Anxiety via Positive and Negative Self-Esteem

First, measurement invariance across gender was examined (1237 girls and 1178 boys). As shown in Table 2, the configural, metric, scalar, and strict invariance models all showed acceptable fit. Changes in RMSEA, CFI, and TLI across the increasingly constrained models remained below the recommended thresholds (ΔRMSEA < 0.015; ΔCFI and ΔTLI < 0.010), supporting measurement invariance across boys and girls.

**Figure 1:**
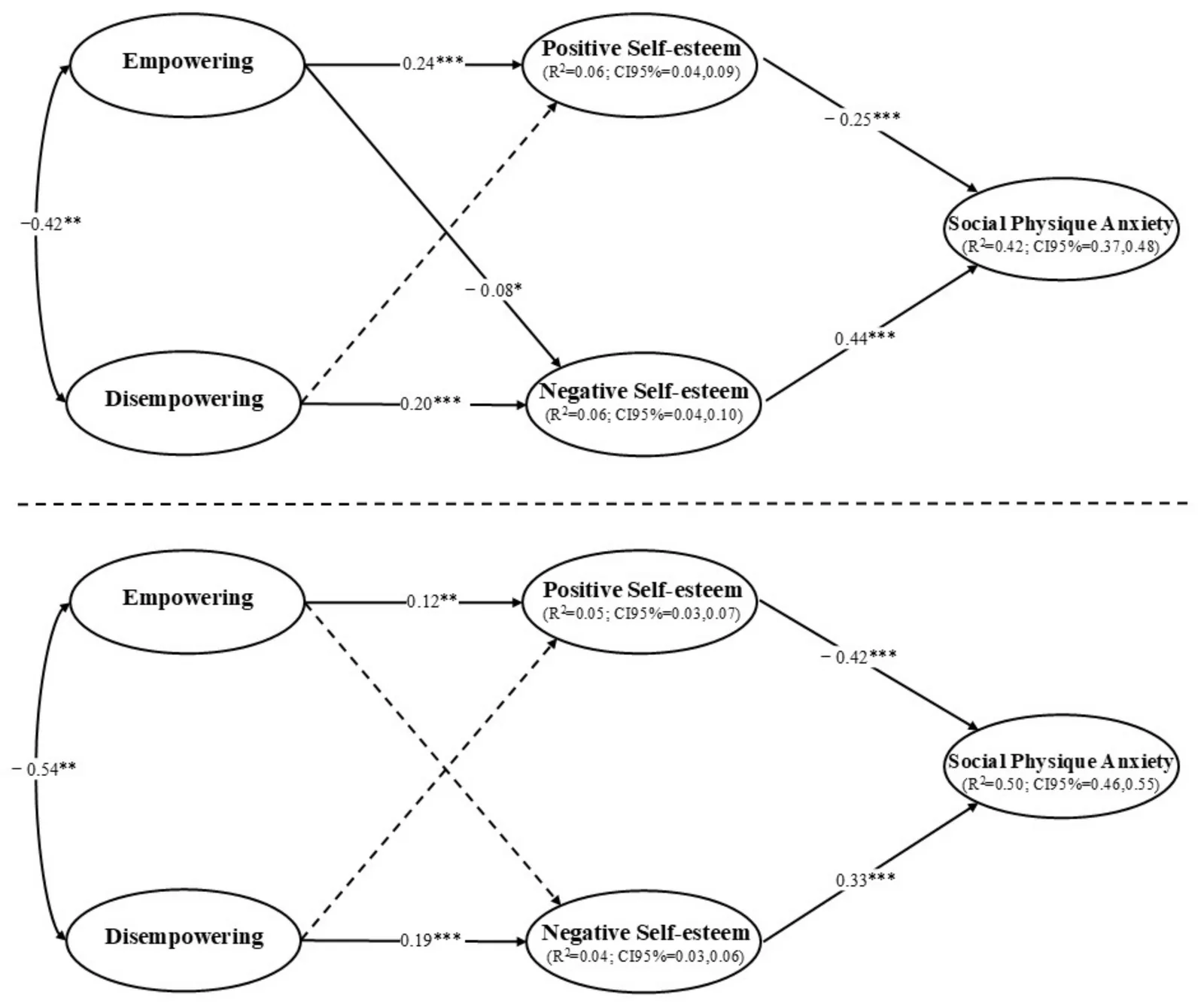
Structural model with latent variables for the associations among (dis-)empowering motivational climate, self-esteem, and SPA. Note: *** *p* < 0.001; ** *p* < 0.01; * *p* < 0.05; R^2^ = explained variance; CI = confidence interval. The 95% CI is reported in parentheses. The dotted arrows represent non-significant relationships. For visual clarity, the direct path from disempowering climate to SPA is not displayed in the Figure 1.

Once the model is demonstrated to be gender-invariant, and the robustness of the measurement model was verified (χ^2^/df = 4.25; *p* < 0.001; CFI = 0.97; TLI = 0.96; SRMR = 0.038; RMSEA = 0.044 (90%CI = 0.042–0.047), the structural model with latent variables (Figure 1) was tested and showed acceptable-to-excellent goodness-of-fit (χ^2^/df = 4.59, *p* < 0.001) [CFI = 0.96; TLI = 0.95; RMSEA = 0.053 (90%CI = 0.050, 0.056); SRMR = 0.048]. The model was adjusted for participants’ age, BMI, and educational centre, and the explained variance reached 42% and 50% for SPA, 6% and 5% for positive self-esteem, and 6% and 4% for negative self-esteem (boys and girls, respectively) (Figure 1). As can be seen, the CI values (95%) of R^2^ are not lower than the minimum values required for interpretation (0.02), so the R^2^ values can be used as ES measurements (Dominguez-Lara, 2017).

Figure 1 shows the direct associations among the variables included in the SEM, specifically the perceived (dis-)empowering climates created by the PE teacher, SPA, and students’ positive and negative self-esteem, analyzed separately for boys and girls.

The empowering climate showed a higher positive coefficient for positive self-esteem in boys than in girls, while positive self-esteem showed a stronger inverse association with SPA in girls than in boys. In contrast, the empowering climate was inversely associated with negative self-esteem among boys, while this association was not significant among girls. Thus, in general, the empowering climate was associated with higher positive self-esteem and lower SPA, and with lower negative self-esteem among boys.

Regarding the disempowering climate, it was positively associated with negative self-esteem among boys and girls (β = 0.20 and 0.19, respectively), while negative self-esteem was positively associated with SPA (β = 0.44 and 0.33, respectively). The cross-sectional indirect association between thedisempowering climate and SPA through negative self-esteem was significant in both groups (β = 0.09 for boys and β = 0.06 for girls). In addition, the direct effect between the disempowering climate and SPA was β = 0.13 for boys and β = 0.07 for girls. Accordingly, the total effects between disempowering climate and SPA were β = 0.22 for boys and β = 0.21 for girls (Table 3).

## 4. Discussion

The purpose of the present study was to examine gender-specific patterns in the associations between perceived teacher-created empowering and disempowering climates and two key outcomes in PE: self-esteem (positive and negative) and SPA. Overall, the findings can be summarised as follows: (a) boys reported significantly higher levels of perceived (dis-)empowering climates as well as higher positive self-esteem than girls; (b) perceiving a more empowering teacher-created climate was associated with higher positive self-esteem, whereas perceiving a more disempowering climate was associated with higher negative self-esteem; moreover, these climate–self-esteem relations were descriptively larger among boys than girls; (c) as expected, positive self-esteem was inversely associated with SPA, whereas negative self-esteem showed a positive association with SPA, with the latter association being descriptively stronger among boys; and (d) a significant association between empowering climate and negative self-esteem emerged only in boys.

Regarding gender mean differences, boys scored higher than girls on perceived teacher-created empowering and disempowering climates and on positive self-esteem. One interpretation is that boys may be more attuned to teacher behaviours and instructional signals in PE, where the teacher’s role is highly salient and classroom cues often carry evaluative meaning (Vasconcellos et al., 2020). This pattern may be explained by gender-linked socialisation and classroom interaction dynamics: boys are frequently encouraged to display greater assertiveness and to engage more actively with authority relationships, which may increase their tendency to notice, anticipate, and respond to teacher directives (Ahmadi et al., 2023). In PE specifically, the competitive and achievement-focused features of many lesson structures may further heighten attention to teacher behaviours that communicate performance standards or interpersonal expectations (Vasconcellos et al., 2020). In addition, the data reflected clear gender differences in self-evaluations: girls reported higher negative self-esteem, whereas boys reported higher positive self-esteem. This aligns with previous evidence indicating lower self-esteem among girls (Hagger & Stevenson, 2010). A reasonable explanation is that adolescent girls may experience stronger body-image concerns, which can undermine self-esteem during this developmental period (Hausenblas et al., 2004). Similarly, girls reported higher SPA, consistent with prior work showing that girls tend to experience greater physique-related anxiety than boys in physical-activity contexts (Hagger & Stevenson, 2010; Granero-Gallegos et al., 2023c). In PE, this may translate into more self-consciousness and greater apprehension about being evaluated by peers, particularly when activities make appearance and performance highly visible (Sicilia et al., 2016). Whereas boys may perceive PE as an opportunity to demonstrate competence and competitiveness, girls may be more likely to interpret the same setting as judgment-oriented, with heightened attention to appearance and public performance (Sicilia et al., 2016).

When we examined the group-specific patterns of association between perceived teacher-created empowering and disempowering climates and students’ self-evaluations and SPA, the pattern was clear: perceptions of both climate types were meaningfully related to self-esteem. In line with Rosenberg’s (1965) conceptualisation of self-esteem as responsive to social evaluation, and consistent with prior evidence in PE (Granero-Gallegos et al., 2023a), a more empowering climate was associated with higher positive self-esteem, whereas a more disempowering climate was associated with higher negative self-esteem. A plausible interpretation is that, when students experience instruction as flexible and choice-supportive, with success defined through self-referenced improvement and with opportunities to build constructive peer connections, they are more likely to appraise themselves positively during PE. By contrast, when teaching is experienced as directive and pressuring, emphasising corrective control and normative comparison to meet predetermined standards, students may be more prone to endorse negative self-esteem. In addition, we observed an inverse association between empowering climate and negative self-esteem. Drawing on Duda’s (2013) framework and related empirical work, this suggests that exposure to empowering teaching may be associated with fewer negative self-evaluative experiences, whereas disempowering cues were not clearly associated with lower positive self-esteem. One possible explanation is that an empowering environment may co-occur with greater perceived competence, social value within the class, and agency over learning.

In addition, we observed that positive self-esteem and SPA were inversely related, whereas negative self-esteem showed a positive association with SPA. This pattern is consistent with the self-presentation perspective (Leary & Kowalski, 1990) and aligns with the limited PE literature reporting comparable links (Granero-Gallegos et al., 2023b; Valero-Valenzuela et al., 2019). A likely interpretation is that students with higher positive self-esteem tend to feel more socially secure in contexts where the body is visible and performance can be evaluated, which may be associated with fewer concerns about appearance-based judgment and lower SPA. In such situations, they may approach participation with greater comfort and a more accepting view of their bodies, making it easier to engage in activities that support skill development and physical competence (Sicilia et al., 2016). By contrast, students characterised by higher negative self-esteem may evaluate their physical selves more harshly and anticipate criticism from others, which may be associated with greater vigilance to social evaluation and higher SPA. As a result, PE participation may become more guarded and self-protective, with heightened attention to external scrutiny and anxiety. Overall, these findings underscore that self-evaluations are relevant to how students interpret and respond to the social demands of PE lessons.

The multigroup SEM yielded different group-specific coefficient patterns among (dis-)empowering climates, self-esteem, and SPA. For boys, an empowering climate showed a stronger association with positive self-esteem, whereas the association between negative self-esteem and SPA was also descriptively stronger among boys. For girls, positive self-esteem showed a stronger inverse association with SPA. These differences in coefficient magnitude should be interpreted descriptively, as they do not by themselves demonstrate statistically significant between-group differences. These results extend prior work suggesting potential gender-differentiated patterns in responses to classroom environments (Abós et al., 2021; Hagger & Stevenson, 2010) and highlight the importance of minimizing disempowering cues and supporting students’ self-esteem in both boys and girls, while considering the higher levels of SPA reported by girls.

### 4.1. Practical Implications

The present findings indicate that teacher-created motivational climates are associated with students’ self-evaluations and appearance-related anxiety, with particularly notable implications for boys’ positive self-esteem during PE lessons. Accordingly, applied efforts should pursue a dual objective: to promote empowering climates that are associated with higher positive self-esteem and lower SPA, and to simultaneously minimise classroom practices that convey disempowering climates and are associated with maladaptive self-presentation experiences (Saiz-González et al., 2024). This dual focus is important because building supportive learning cues is unlikely to be sufficient if pressuring, comparison-oriented signals remain present in everyday instruction. To translate these insights into practice, teacher education and continuing professional development should explicitly address how specific instructional behaviours are associated with students’ self-presentation concerns (Ocete et al., 2025). In particular, training initiatives should increase teachers’ awareness of the potential risks for SPA associated with disempowering climates, while emphasising the pedagogical value of empowering climates as a supportive context. Such programmes should provide PE teachers with concrete, observable strategies to implement autonomy-supportive, task-involving, and social-supportive teaching approaches (e.g., offering meaningful options, using informational feedback, structuring activities around personal improvement criteria, and promoting respectful peer interaction), alongside guidance on identifying and replacing practices that reflect disempowering climates (e.g., coercive language, excessive corrective control, and evaluation routines that prioritise normative ranking) (Méndez-Giménez & Garví-Medrano, 2025; Rojo-Ramos et al., 2024a; Ruiz-Montero et al., 2025). Given the gender-differentiated patterns observed in this study, PE teachers should aim to maintain high levels of an empowering climate and keep disempowering cues to a minimum for both girls and boys, while paying particular attention to how these cues may differentially relate to students’ self-esteem and SPA. In parallel, lesson design can be used to actively cultivate positive self-esteem by foregrounding individual progress, recognising effort and mastery, and delivering feedback that is improvement-focused rather than comparison-based. These practices are intended to support students’ confidence in their physical capabilities and address concerns about negative appearance-related evaluation. Ultimately, greater sensitivity to gender-specific responses to (dis-)empowering climates should enable educators to create PE environments that more consistently support adaptive self-perceptions and address anxiety linked to physical self-presentation.

### 4.2. Limitations and Future Research

Despite the above findings, the present research also has certain limitations. First, employing a convenience sampling approach limits the ability to extrapolate the findings to a wider population. Therefore, further research is needed to examine these results in samples of students across different school levels (e.g., higher education), school types (e.g., private schools), and diverse cultural contexts. Second, the main findings of this research in physical education are based only on student self-report questionnaires. Although students’ perceptions of (dis-)empowering climates and self-presentation are central to our analysis, future research should incorporate a multi-informant approach, including input from teachers, external observers and students, to more thoroughly assess how these educational climates are fostered by teachers and their associations with students’ psychological states. Third, although this study was grounded in Duda’s (2013) theoretical framework, the cross-sectional design does not allow causal or temporal relationships to be established between perceived (dis-)empowering climates and students’ self-presentation outcomes. Moreover, estimating indirect effects from cross-sectional data may introduce bias because the temporal ordering among the variables cannot be determined. Therefore, the indirect associations observed in this study should be interpreted cautiously and not as evidence of a causal mediation process. Additional longitudinal studies are needed to examine the temporal sequence among perceived (dis-)empowering climates, self-esteem, and self-presentation-related outcomes in physical education. Fourth, the nested structure of students within classes, teachers, and schools was not explicitly accounted for in the SEM, which may have affected the estimation of standard errors. Future studies should consider multilevel modelling or cluster-robust estimation procedures. Moreover, further studies are required to expand the nomological network of (dis-)empowering climates by analyzing new self-related outcomes. This would allow one to gain a better understanding of the role that teacher-created (dis-)empowering climates play in the PE lesson.

## 5. Conclusions

This study adds evidence to a small body of research in the PE field, demonstrating the existence of gender differences in students’ perceptions of (dis-)empowering climates, self-esteem and SPA. Taken together, the results of this study suggest that both disempowering and empowering climates were associated with boys’ and girls’ self-presentation experiences in PE, with the (dis-)empowering climate positively associated with negative self-esteem in both groups. Additionally, this study highlights the relevance of the associations among empowering climate, positive self-esteem, and lower SPA. Overall, the results suggest that training programs for PE pre-service teachers and ongoing professional development for in-service teachers should focus on strategies to minimize disempowering practices and develop empowering interactions with their students.

## Figures and Tables

**Table 1:** Descriptive statistics, means differences by gender, and latent correlations.

	Girls (*n* = 1237)		Boys (*n* = 1178)		Mean Differences				Correlations				
*Variables*	*M*	*SD*	*M*	*SD*	Dif	*t*(df)	*p*-Value	*d*	1	2	3	4	5
1. Empowering	3.73	0.82	3.82	0.73	−0.09	−2.85(2402) ^a^	0.005	0.16	−	−0.41 ***	0.18 ***	−0.14 ***	−0.11 ***
2. Disempowering	2.13	0.82	2.35	0.86	−0.22	−6.45(2413)	<0.001	0.26	−0.51 ***	−	−0.06 *	0.20 ***	0.19 ***
3. Positive self-esteem	3.04	0.61	3.31	0.58	−0.27	−10.99(2413)	<0.001	0.45	0.14 ***	−0.10 ***	−	−0.42 ***	−0.41 ***
4. Negative self-esteem	2.30	0.81	2.04	0.83	0.26	7.66(2413)	<0.001	0.31	−0.07 ***	0.15 ***	−0.55 ***	−	0.55 ***
5. Social physique anxiety	2.87	1.09	2.26	0.98	0.61	14.44(2403) ^a^	<0.001	0.59	−0.04	0.10 ***	−0.51 ***	0.60 ***	−

Note: M = mean; SD = standard deviation; Dif = mean difference; df = degrees of freedom; d = Cohen’s d; ^a^ = Levene’s test is significant, the variances are not equal. Numbers above diagonal display correlations for boys; numbers below diagonal show correlations for girls. *** *p* < 0.001; * *p* < 0.05.

**Table 2:** Gender invariance analysis by gender.

Model	χ^2^	df	RMSEA [90% CI]	CFI	TLI	Model Comparison	ΔRMSEA	ΔCFI	ΔTLI
1.- M1	1393.89 **	416	0.031 [0.029–0.033]	0.965	0.965	-			
2.- M2	1444.23 **	432	0.031 [0.029–0.033]	0.963	0.963	2 versus 1	0.000	−0.002	−0.002
3.- M3	1475.70 **	446	0.031 [0.029–0.033]	0.961	0.963	3 versus 2	0.000	−0.002	0.000
4.- M4	1529.38 **	456	0.031 [0.030–0.033]	0.957	0.961	4 versus 3	0.000	−0.004	−0.002

Note. χ^2^ = Chi square; df = degrees of freedom; RMSEA = Root Mean Square Error of Approximation; 90%CI = 90% confidence interval of RMSEA; CFI = Comparative Fit Index; TLI Tucker–Lewis Index; ** *p* < 0.001; M1 = Model 1, unrestricted; M2 = Model 2, restricted in factor loadings; M3 = Model 3, restricted in factor loadings and intercepts; M4 = Model 4, restricted in factor loadings, intercepts, and error variances.

**Table 3:** Standardized direct, cross-sectional indirect, and total associations in the structural model.

Independent Variable	Dependent Variable	Mediator	β	SE	95%CI	
					Lower	Upper
Direct effects						
Empowering	Positive self-esteem		0.24 *** (0.12 ***)	0.02(0.02)	0.17(0.04)	0.30(0.18)
Empowering	Negative self-esteem		−0.08 * (0.02)	0.02(0.02)	−0.16(−0.04)	−0.03(0.08)
Disempowering	Negative self-esteem		0.20 *** (0.19 ***)	0.03(0.03)	0.13(0.14)	0.29(0.30)
Empowering	SPA		0.09 ** (0.11 *)	0.03(0.03)	0.05(0.03)	0.15(0.15)
Disempowering	SPA		0.13 ** (0.07)	0.04(0.03)	0.08(0.05)	0.20(0.12)
Positive self-esteem	SPA		−0.25 *** (−0.43 ***)	0.05(0.06)	−0.16(−0.32)	−0.34(−0.49)
Negative self-esteem	SPA		0.44 *** (0.33 ***)	0.05(0.05)	0.36(0.25)	0.54(0.44)
Indirect effects						
Empowering	SPA	Positive self-esteem	−0.06 *** (−0.05 ***)	0.01(0.01)	−0.08(−0.07)	−0.03(−0.02)
Empowering	SPA	Negative self-esteem	−0.04* (0.01)	0.02(0.02)	−0.08(−0.04)	0.03(0.04)
Disempowering	SPA	Negative self-esteem	0.09 ** (0.06 ***)	0.02(0.02)	0.05(0.04)	0.12(0.09)
Total effects						
Empowering	SPA		0.00(0.12)	0.03(0.04)	−0.06(−0.01)	0.06(0.12)
Disempowering	SPA		0.22 * (0.21 **)	0.04(0.04)	0.15(0.08)	0.30(0.31)

Note. β = standardized estimate; SE = standard error; SPA = social physique anxiety; 95% CI = 95% confidence interval; Lower = 95% CI lower limit; Upper = 95% CI upper limit; *** *p* < 0.001; ** *p* < 0.01; * *p* < 0.05. Girls’ scores are reported in parentheses.

## Data Availability

The data are available from the corresponding author upon reasonable request.
